# Dental Calculus Stimulates Interleukin-1β Secretion by Activating NLRP3 Inflammasome in Human and Mouse Phagocytes

**DOI:** 10.1371/journal.pone.0162865

**Published:** 2016-09-15

**Authors:** Jorge Luis Montenegro Raudales, Atsutoshi Yoshimura, Ziauddin SM, Takashi Kaneko, Yukio Ozaki, Takashi Ukai, Toshihiro Miyazaki, Eicke Latz, Yoshitaka Hara

**Affiliations:** 1 Department of Periodontology, Graduate School of Biomedical Sciences, Nagasaki University, Nagasaki, Japan; 2 Center for Oral Diseases, Fukuoka Dental College, Fukuoka, Japan; 3 Department of Cell Biology, Graduate School of Biomedical Sciences, Nagasaki University, Nagasaki, Japan; 4 University Hospital, University of Bonn, Bonn, Germany; 5 Department of Infectious Diseases and Immunology, University of Massachusetts Medical School, Worcester, Massachusetts, United States of America; 6 German Center for Neurodegenerative Diseases, Bonn, Germany; 7 Centre of Molecular Inflammation Research, Norwegian University of Science and Technology, Trondheim, Norway; Toho Daigaku, JAPAN

## Abstract

Dental calculus is a mineralized deposit associated with periodontitis. The bacterial components contained in dental calculus can be recognized by host immune sensors, such as Toll-like receptors (TLRs), and induce transcription of proinflammatory cytokines, such as IL-1β. Studies have shown that cellular uptake of crystalline particles may trigger NLRP3 inflammasome activation, leading to the cleavage of the IL-1β precursor to its mature form. Phagocytosis of dental calculus in the periodontal pocket may therefore lead to the secretion of IL-1β, promoting inflammatory responses in periodontal tissues. However, the capacity of dental calculus to induce IL-1β secretion in human phagocytes has not been explored. To study this, we stimulated human polymorphonuclear leukocytes (PMNs) and peripheral blood mononuclear cells (PBMCs) with dental calculus collected from periodontitis patients, and measured IL-1β secretion by ELISA. We found that calculus induced IL-1β secretion in both human PMNs and PBMCs. Calculus also induced IL-1β in macrophages from wild-type mice, but not in macrophages from NLRP3- and ASC-deficient mice, indicating the involvement of NLRP3 and ASC. IL-1β induction was inhibited by polymyxin B, suggesting that LPS is one of the components of calculus that induces pro-IL-1β transcription. To analyze the effect of the inorganic structure, we baked calculus at 250°C for 1 h. This baked calculus failed to induce pro-IL-1β transcription. However, it did induce IL-1β secretion in lipid A-primed cells, indicating that the crystalline structure of calculus induces inflammasome activation. Furthermore, hydroxyapatite crystals, a component of dental calculus, induced IL-1β in mouse macrophages, and baked calculus induced IL-1β in lipid A-primed human PMNs and PBMCs. These results indicate that dental calculus stimulates IL-1β secretion via NLRP3 inflammasome in human and mouse phagocytes, and that the crystalline structure has a partial role in the activation of NLRP3 inflammasome.

## Introduction

Periodontitis is an inflammatory disease that leads to the destruction of the tissues surrounding the teeth. The release of inflammatory mediators such as prostaglandins, matrix metalloproteinases, and cytokines, promotes tissue damage [1]. The pro-inflammatory cytokine interleukin (IL)-1β is one of the main factors in the inflammatory process, since it affects nearly all cell types and is involved in bone resorption [2, 3]. Higher levels of IL-1β are detected in the gingival crevicular fluid (GCF) in sites affected by periodontitis, relative to GCF from healthy sites [4, 5]. IL-1β levels in gingival tissues and GCF correlates with the inflammatory status of periodontal disease, indicating the fundamental role of IL-1β in the pathogenesis of periodontitis [6].

IL-1β production is regulated both transcriptionally and post-transcriptionally [7]. The transcription of pro-IL-1β can be triggered by any stimulus that initiates receptor signaling, which activates nuclear factor (NF)-κB. Microbial ligands activate NF-κB through Toll-like receptors (TLRs) and NOD-like receptors (NLRs), while cytokines, such as TNF-α and IL-1β, activate signaling through their own receptors [8]. Pro-IL-1β is biologically inactive and can be processed into its active form upon activation of the intracellular multiprotein complex known as inflammasome [9]. Inflammasomes consist of a sensor molecule, such as NLR, the apoptosis speck-like protein containing a caspase-recruitment and activation domain (ASC), and pro-caspase-1. Assembly of the inflammasome induces the autocatalysis of pro-caspase-1 into active caspase-1, which cleaves pro-IL-1β to its mature form [10]. The role of the NLRP3 inflammasome in periodontitis has recently been investigated [11, 12]. These studies showed that NLRP3, but not ASC, was expressed at significantly higher levels in gingival tissue from patients suffering from gingivitis or periodontitis than in that from healthy individuals. In addition, there was a positive correlation between NLRP3 and IL-1β expression levels in these tissues, confirming the involvement of NLRP3 inflammasome in the pathogenesis of periodontitis.

Dental calculus is a mineralized deposit frequently found in periodontal pockets, 70–80% of which comprises inorganic structures [13]. Calculus usually develops after plaque formation [14]. Small crystals appear in the intermicrobial matrix, often in close proximity to the bacteria. The matrix between the microorganisms is gradually calcified, followed by mineralization of the bacteria. Calcium phosphate is the main crystal form found, including hydroxyapatite (HA), brushite, whitlockite or tricalcium phosphate, and octacalcium phosphate [13, 15]. The remaining organic matrix is made up of proteins, desquamated epithelial cells, leukocytes, and microorganisms [16].

Since dental calculus is always covered by viable bacterial plaque, it is difficult to distinguish its effects on periodontal tissues, and has been regarded as a plaque retention factor [16, 17]. However, a number of cross-sectional and longitudinal epidemiological studies have demonstrated a clear association between calculus deposition and periodontitis [16]. In addition, retained subgingival calculus increases inflammation following flap surgery [18]. Recent evidence has shown that basic calcium phosphate crystals, including HA, can induce IL-1β secretion in human monocytes and macrophages primed with lipopolysaccharide (LPS) [19]. Basic calcium phosphate crystals stimulate NLRP3 inflammasome activation through phagocytosis, in the same manner as silica, calcium pyrophosphate dehydrate, monosodium urate (MSU), and cholesterol crystals [20–22]. Electron microscopy of dental calculus has revealed the presence of minute crystals, 500–4000 Å long, deposited upon mineralized microorganisms [23]. We therefore hypothesized that dental calculus particles can be incorporated by the phagocytes in GCF and might induce IL-1β secretion. Although the common periodontal pathogens in dental plaque, *Porphyromonas gingivalis* and *Treponema denticola*, activate NLRP3 inflammasome in human macrophages [24, 25], dental calculus might contribute to inflammation in gingival tissue by further activating NLRP3 inflammasome.

The aim of this study was to determine if dental calculus can induce IL-1β secretion in human and mouse phagocytes. We found that dental calculus contains components that can induce pro-IL-1β transcription, and that its crystalline structure can activate NLRP3 inflammasome to induce secretion of mature IL-1β in human and mouse phagocytes.

## Materials and Methods

### Dental calculus

Samples of dental calculus were obtained from five chronic periodontitis patients who had visited the Nagasaki University Hospital. Written informed consent was obtained from all participants. The design of the study and procedures for obtaining informed consent were approved by the Institutional Review Board at Nagasaki University. Each dental calculus sample was pulverized and treated overnight with 10% sodium hypochlorite to remove the organic components [13, 26, 27], and washed three times with distilled water. The calculus was then filtered using a 48 μm nylon mesh and autoclaved. These samples were termed unbaked calculus. To remove microbial remnants, some of the unbaked calculus was treated at 250°C for 1 h [28], and these samples were termed baked calculus. This heat treatment has been reported to effectively destroy pyrogenic substances, such as endotoxin [29]. All calculus samples thus prepared were weighed, adjusted to the appropriate concentrations, and vigorously vortexed before use for assessing cell stimulation.

### Reagents

Dulbecco’s modified Eagle’s medium (DMEM), RPMI 1640 cell culture media, PBS, penicillin-streptomycin, and RNase H were purchased from Thermo Fisher Scientific (Waltham, MA). Fetal bovine serum (FBS) was obtained from Equitec-Bio (Kerrville, TX). Synthetic HA crystals, ≤ 5 μm in diameter, were purchased from SofSera (Tokyo, Japan). DMSO, polymyxin B, MCC950 and glyburide were purchased from Sigma-Aldrich (St. Louis, MO). Caspase-1 inhibitor, z-YVAD-fmk, was obtained from Calbiochem-EMD Millipore (Darmstadt, Germany). An actin-polymerization inhibitor, cytochalasin D, was purchased from Wako Pure Chemical Industries (Osaka, Japan). Ultra-pure LPS from *E*. *coli* O111:B4 was obtained from InvivoGen (San Diego, CA). Mono-Poly resolving medium was purchased from MP Biomedicals (Santa Ana, CA). Human and mouse IL-1β DuoSet enzyme-linked immunosorbent assay (ELISA) kit was purchased from R&D Systems (Minneapolis, MN). An RNeasy Mini Kit and a QIAprep Spin Miniprep Kit were obtained from QIAGEN (Hilden, Germany). Avian myeloblastosis virus reverse transcriptase and CytoTox 96 Non-Radioactive Cytotoxicity Assay was purchased from Promega (Madison, WI). Primers for mouse IL-1β and glyceraldehydes-3-phosphate-dehydrogenase (GAPDH) genes were obtained from Hokkaido System Science (Sapporo, Japan). SYBR *Premix Ex Taq* (Tli RNase H Plus) was obtained from Takara Bio (Otsu, Japan). iPGell was purchased from GenoStaff (Tokyo, Japan). Synthetic lipid A (compound 506) was acquired from the Peptide Institute (Osaka, Japan).

### Cell isolation and culture

Human PMNs and PBMCs were isolated from the peripheral blood of three systemically and periodontally healthy donors recruited from Nagasaki University. The collection of PMNs and PBMCs was approved by the Institutional Review Board at Nagasaki University. All donors received and completed a written informed consent form prior to participation in the study. The cells were separated using Mono-Poly resolving medium, resuspended in RPMI 1640 medium supplemented with 10% FBS, 100 U/mL penicillin, and 100 μg/mL streptomycin. Immortalized bone marrow macrophages from wild-type (C57BL/6), NLRP3-deficient, and ASC-deficient mice, were obtained from the Institute of Innate Immunity, University Hospitals Bonn, Germany [20] and cultured in DMEM supplemented with 10% FBS, 100 U/mL penicillin and 100 μg/mL streptomycin.

### Measurement of IL-1β

Human PMNs, PBMCs, or mouse macrophages (1 × 10^5^ cells) were seeded in 96-well plates, then stimulated with unbaked or baked calculus for 6–8 h. To evaluate the effect of the crystalline structure, human PMNs and PBMCs were primed with 1 ng/mL lipid A for 2 h. Mouse macrophages were primed with 200 ng/mL lipid A or 100 ng/mL ultra-pure LPS for 2 h. The primed and unprimed cells were then stimulated with baked calculus or HA particles for 6–8 h. For the inhibition assays, the primed or unprimed cells were pre-incubated with polymyxin B (LPS inhibitor), cytochalasin D (actin polymerization inhibitor), z-YVAD-fmk (caspase-1 inhibitor), MCC950 (NLRP3 inhibitor) [30] or glyburide (NLRP3 inhibitor, ATP-sensitive K^+^ channel blocker) [31] for 30 min before stimulation with unbaked calculus, baked calculus or HA particles. The culture supernatants were harvested and the concentrations of IL-1β quantified by ELISA, according to the manufacturer´s protocol.

### Cytotoxicity assay

Human PMNs, PBMCs or mouse macrophages (1 × 10^5^ cells) were seeded in 96-well plates and stimulated with unbaked or baked calculus for 6–8 h. The 96-well plates were centrifuged at 250 × *g* for 4 min and 50 μl cell culture supernatants were harvested. Then, lactate dehydrogenase (LDH) in the supernatants was measured using a CytoTox 96 Non-Radioactive assay according to the manufacturer's protocol. Percent cytotoxicity was determined according to the following calculation [32]:

$$\%\;Cytotoxicity=100\times \;\frac{\left(Experimental-Culture\;Medium\;Background\right)}{\left(Maximun\;LDH\;Release-Culture\;Medium\;Background\right)}$$ (1)

### Quantitative reverse transcription PCR (RT-qPCR)

For analysis of IL-1β mRNA levels, mouse macrophages (3 × 10^5^ cells) from wild-type, NLRP3-deficient, and ASC-deficient mice were seeded in 24-well plates and stimulated with 500 μg/mL unbaked or baked calculus for the indicated time periods. After stimulation, total RNA was extracted from the cells using the RNeasy Mini Kit with on-column DNase treatment according to the manufacturer’s protocol. For each sample, 200 ng of total RNA was converted to first strand cDNA, using avian myeloblastosis virus reverse transcriptase, at 25°C for 10 min, followed by 50 min at 42°C, and 15 min at 70°C, with a Takara PCR thermo cycler (Takara Bio). The cDNA was treated with RNase H and purified using the QIAprep Spin Miniprep Kit. The primer sequences used were as follows: IL-1β forward 5′-GGAGAACCAAGCAACGACAAAATA-3′ and reverse, 5′-TGGGGAACTCTGCAGACTCAAAC-3′; GAPDH forward, 5′-GGAGGAACCTGCCAAGTATG-3′, and reverse, 5′-TGGGAGTTGCTGTTGAAGTC-3′ [33]. PCR comparative quantification was performed with SYBR *Premix Ex Taq* using the Mx3000P qPCR System (Agilent technologies, Santa Clara, CA). The amplification conditions were: 95°C for 10 sec, followed by 40 cycles of 95°C for 5 sec, 55°C for 20 sec, and a final cycle of 95°C for 1 min, 55°C for 30 sec, 95°C for 30 sec. Melting curve analysis was used to confirm that the proper PCR products were amplified in all samples. The IL-1β mRNA relative expression ratio was calculated based on PCR efficiency and the threshold cycle difference for the test sample (stimulated cells) versus a calibrator (unstimulated cells). Target gene expression was normalized by GAPDH gene expression [34]. IL-1β mRNA levels of the calibrator were set to 1.

### Microscopic analyses

Macrophages (3 × 10^5^) from wild-type mice were seeded in 48-well plates. After overnight incubation, cells were pretreated with 0, 1 or 7.5 μM of cytochalasin D for 30 min and then incubated with unbaked calculus for 8 h. The cells were immobilized in iPGell and fixed in 0.05 M cacodylate buffer (pH7.4) containing 2.5% glutaraldehyde and 2% paraformaldehyde, post-fixed with osmium tetraoxide in 0.05 M cacodylate buffer (pH7.4), and embedded in Epon-Araldite resin. For analysis of the uptake of dental calculus in the macrophages, semithin sections (1 μm) were stained with toluidine blue and examined with a Zeiss Axioskop 2 light microscope (Zeiss, Oberkochen, Germany) at 1000 times magnification. Ultrathin sections (0.1 μm) were stained with 2% uranyl acetate and Reynold’s lead citrate, and examined under a Hitachi H-7100 electron microscope (Hitachi High-Technologies, Tokyo, Japan).

### Statistical Analysis

The statistical differences between the test and control groups were analyzed by means of *t*-tests, using Stat Mate III (ATMS, Tokyo, Japan).

## Results

### Unbaked calculus induces IL-1β secretion via NLRP3-inflammasome in human PMNs and PBMCs

**Fig 1:**
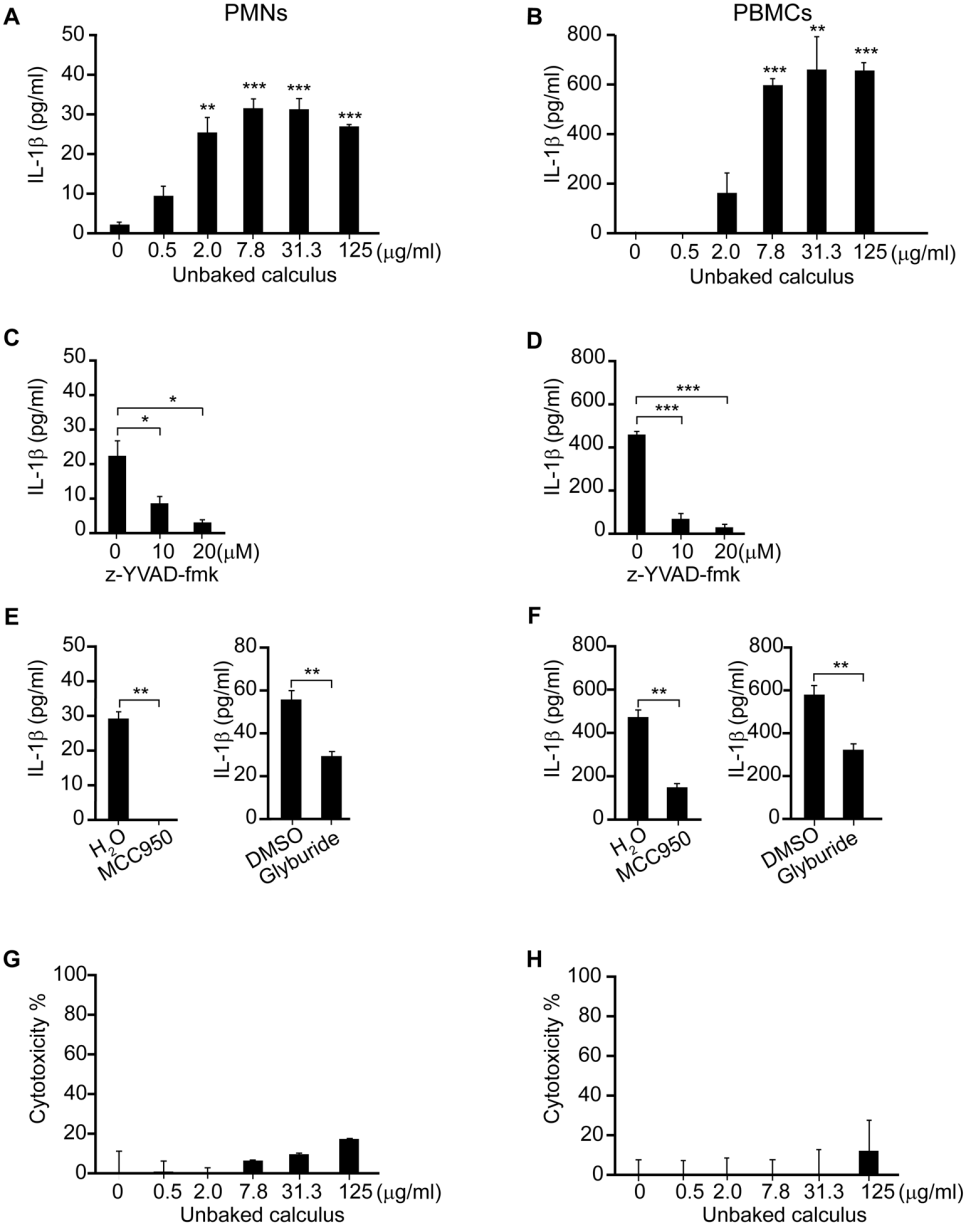
Unbaked dental calculus induces IL-1β secretion via NLRP3-inflammasome in human PMNs and PBMCs. Freshly isolated human PMNs (A, G) and PBMCs (B, H) were stimulated with indicated concentrations of unbaked calculus. PMNs (C, E) and PBMCs (D, F) were also stimulated with 125 μg/mL of unbaked calculus in the presence or absence of the caspase-1 inhibitor, z-YVAD-fmk (10 or 20 μM) (C, D), MCC950 (10 μM) or glyburide (25 μM) (E, F). IL-1β concentrations in the culture supernatants were measured by ELISA (A–F). Cytotoxicity was quantified by measuring LDH release in the culture supernatants (G, H). The results are expressed as the mean ± standard error of triplicate assays. Representative results of three independent experiments using different donors are shown. *p < 0.05; **p < 0.01; ***p < 0.001 (*t*-tests).

To explore the capacity of dental calculus to induce IL-1β secretion, freshly isolated human PMNs and PBMCs were exposed to increasing concentrations of unbaked calculus or PBS for 6 h. Unbaked calculus induced a dose-dependent release of IL-1β in human PMNs (Fig 1A) and PBMCs (Fig 1B). To examine whether this induction was dependent on NLRP3 inflammasome, human PMNs and PBMCs were stimulated with unbaked calculus with or without preincubation with the caspase-1 inhibitor, z-YVAD-fmk (10 or 20 μM) or the NLRP3 inhibitors, MCC950 (10 μM) or glyburide (25 μM). IL-1β secretion was significantly reduced by all of these inhibitors in both PMNs (Fig 1C and 1E) and PBMCs (Fig 1D and 1F). This suggested that unbaked calculus induces IL-1β secretion via the activation of NLRP3 inflammasome in human PMNs and PBMCs. A recent study has suggested that human monocytes secrete IL-1β via an alternative inflammasome pathway without causing cell death in response to LPS [35]. We examined whether human PMNs and PBMCs secreted IL-1β with or without cell death. Only high concentrations of unbaked calculus induced cell death in both human PMNs (Fig 1G) and PBMCs (Fig 1H), suggesting that cell death was not required for IL-1β secretion induced by unbaked calculus in these cell types.

### Unbaked calculus induces NLRP3 inflammasome-dependent IL-1β secretion in mouse macrophages

**Fig 2:**
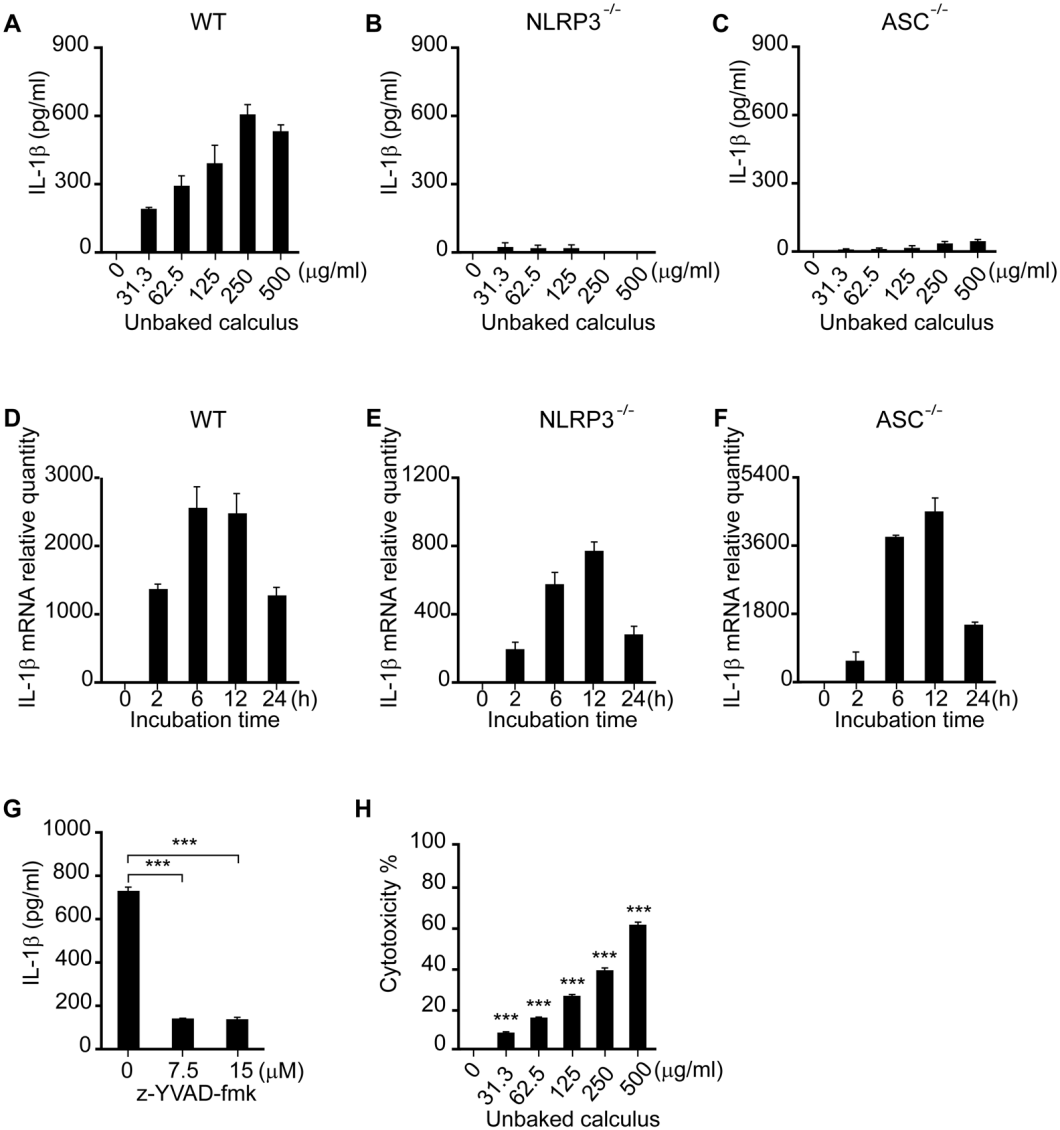
NLRP3-, ASC- and caspase-1-dependent secretion of IL-1β in mouse macrophages stimulated with unbaked calculus. Macrophages from wild-type (WT) (A, D, G, H), NLRP3-deficient (B, E), and ASC-deficient (C, F) mice were stimulated with indicated concentrations (A–C, H) or 500 μg/mL (D–G) of unbaked calculus for 8 h (A–C, G, H) or the indicated time (D–F). In G, the cells were pre-incubated with the caspase-1 inhibitor, z-YVAD-fmk. IL-1β concentrations in the culture supernatants were measured by ELISA (A–C, G). Total RNA was extracted and the relative quantity of IL-1β mRNA determined by RT-qPCR. The IL-1β mRNA levels were normalized to GAPDH and the expression levels at 0 h were adjusted to 1 (D–F). Cytotoxicity was quantified by measuring LDH release in the culture supernatants (H). The results are expressed as the mean ± standard error of triplicate assays. Representative results of three independent experiments are shown. ***p < 0.001 (*t*-tests).

To confirm which inflammasome components are involved in IL-1β secretion, immortalized bone marrow macrophages from wild-type, NLRP3-deficient and ASC-deficient mice were stimulated with increasing doses of unbaked calculus for 8 h. IL-1β was released in a dose-dependent fashion in macrophages from wild-type mice (Fig 2A), whereas only low levels of IL-1β secretion were detected in those from NLRP3-deficient and ASC-deficient mice (Fig 2B and 2C), suggesting that NLRP3, as well as its adaptor protein ASC, are essential for IL-1β release.

To examine the priming status, we measured pro-IL-1β mRNA levels in macrophages stimulated with 500 μg/mL of unbaked calculus. Unbaked calculus up-regulated pro-IL-1β mRNA expression in macrophages from all three types of mice, confirming intact priming in each cell line (Fig 2D–2F). To further assess the involvement of caspase-1, mouse macrophages were stimulated with 500 μg/mL of unbaked calculus in the presence or absence of z-YVAD-fmk (7.5 or 15 μM). As can be seen in Fig 2G, z-YVAD-fmk significantly inhibited IL-1β secretion in mouse macrophages. These results show that the unbaked calculus induced IL-1β secretion via the activation of NLRP3 inflammasome in mouse macrophages.

Contrary to human PMNs and PBMCs, murine cell lines undergo cell death after the activation of the NLRP3 inflammasome [35]. To investigate if stimulation with unbaked calculus leads to cell death, macrophages from wild-type mice were stimulated with increasing doses of unbaked calculus. After 8 h of incubation, LDH was released in a dose-dependent manner (Fig 2H). These results suggest that unbaked calculus induced the classical NLRP3 activation in murine macrophages.

**Fig 3:**
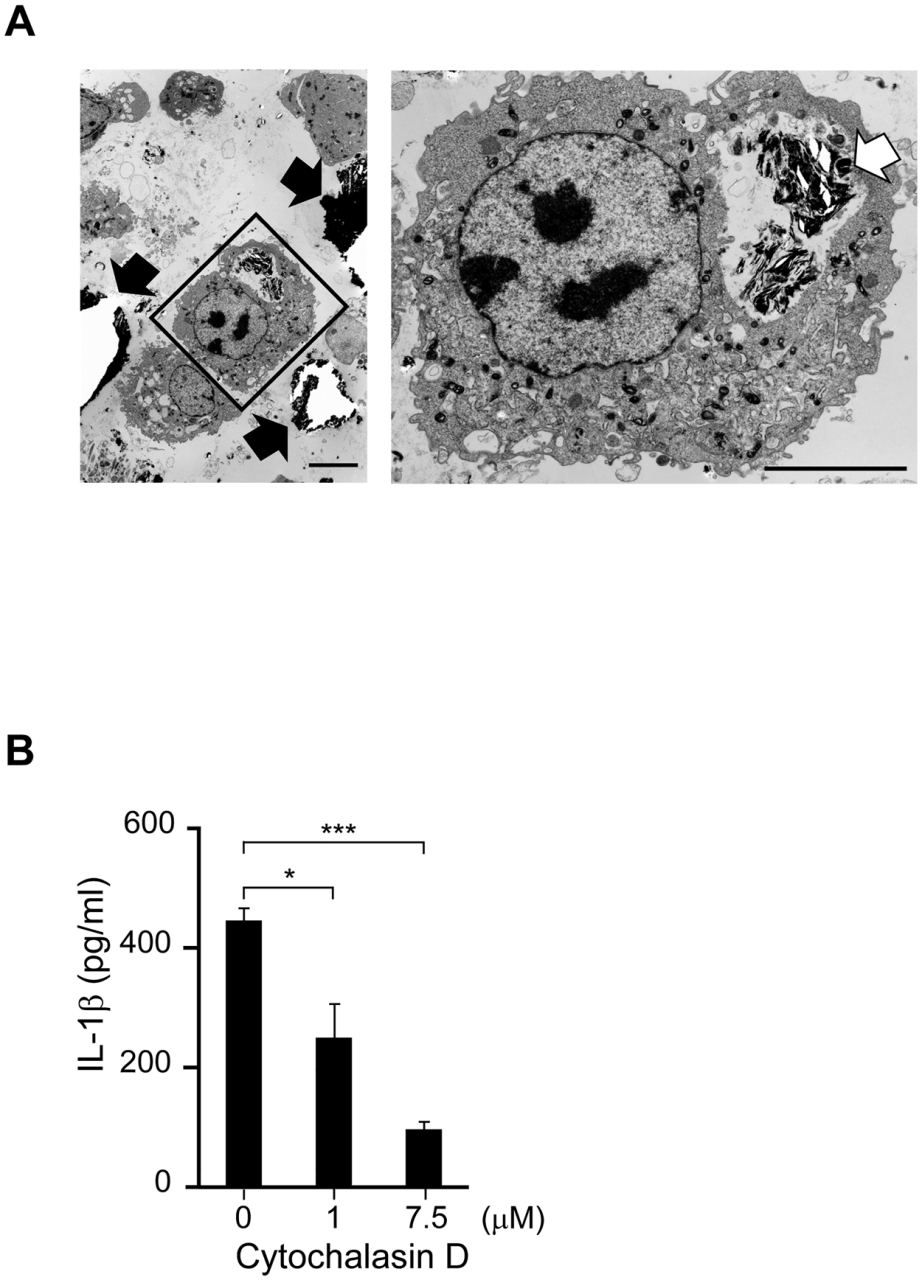
IL-1β secretion induced by unbaked calculus was suppressed by phagocytosis inhibitor. Macrophages from wild-type mice were left untreated (A) or pre-incubated with the indicated concentrations of the phagocytosis inhibitor cytochalasin D (B). The cells were then stimulated with 500 μg/mL of unbaked calculus for 8 h. Low- (left) and high-magnification (right) electron microscope images are shown (A). The white arrow marks the location of the unbaked calculus inside the cells, and black arrows mark the location of the unbaked calculus outside the cells. Scale bars: 5 μm. IL-1β concentrations in the culture supernatants were measured by ELISA (B). The results are expressed as the mean ± standard error of triplicate assays. Representative results of three independent experiments are shown. *p < 0.05; ***p < 0.001 (*t*-tests).

Numerous stimuli have been identified as activators of the NLRP3 inflammasome, including crystalline particles that require phagocytosis for activation [19–21], adenosine triphosphate (ATP), which acts through the cell surface receptor P2X7R [36], and the pore-forming toxin, nigericin [8]. To examine whether the induction of IL-1β by unbaked calculus is dependent on phagocytosis, we stimulated mouse macrophages with 500 μg/ml of unbaked calculus in the presence or absence of an inhibitor of actin-polymerization, cytochalasin D (1 or 7.5 μM) for 8 h. In the absence of cytochalasin D, the cells incorporated calculus particles that possessed a high-electron-density crystal structure (Fig 3A), as described previously [37]. Relatively high levels of IL-1β secretion were induced in the absence of cytochalasin D; however, IL-1β secretion was inhibited by cytochalasin D (Fig 3B). Concomitantly, light microscopy images showed that cytochalasin D reduced the number of cells incorporating calculus-like particles (S1A–S1C Fig), and this reduced phagocytosis of calculus was confirmed by electron microscope imaging (S1D Fig). These results indicate that IL-1β secretion is mediated by phagocytosis, and that the crystalline particles found in unbaked calculus are likely stimuli for inflammasome activation.

### Crystalline structures in dental calculus contribute to IL-1β secretion in mouse macrophages and human PMNs and PBMCs

**Fig 4:**
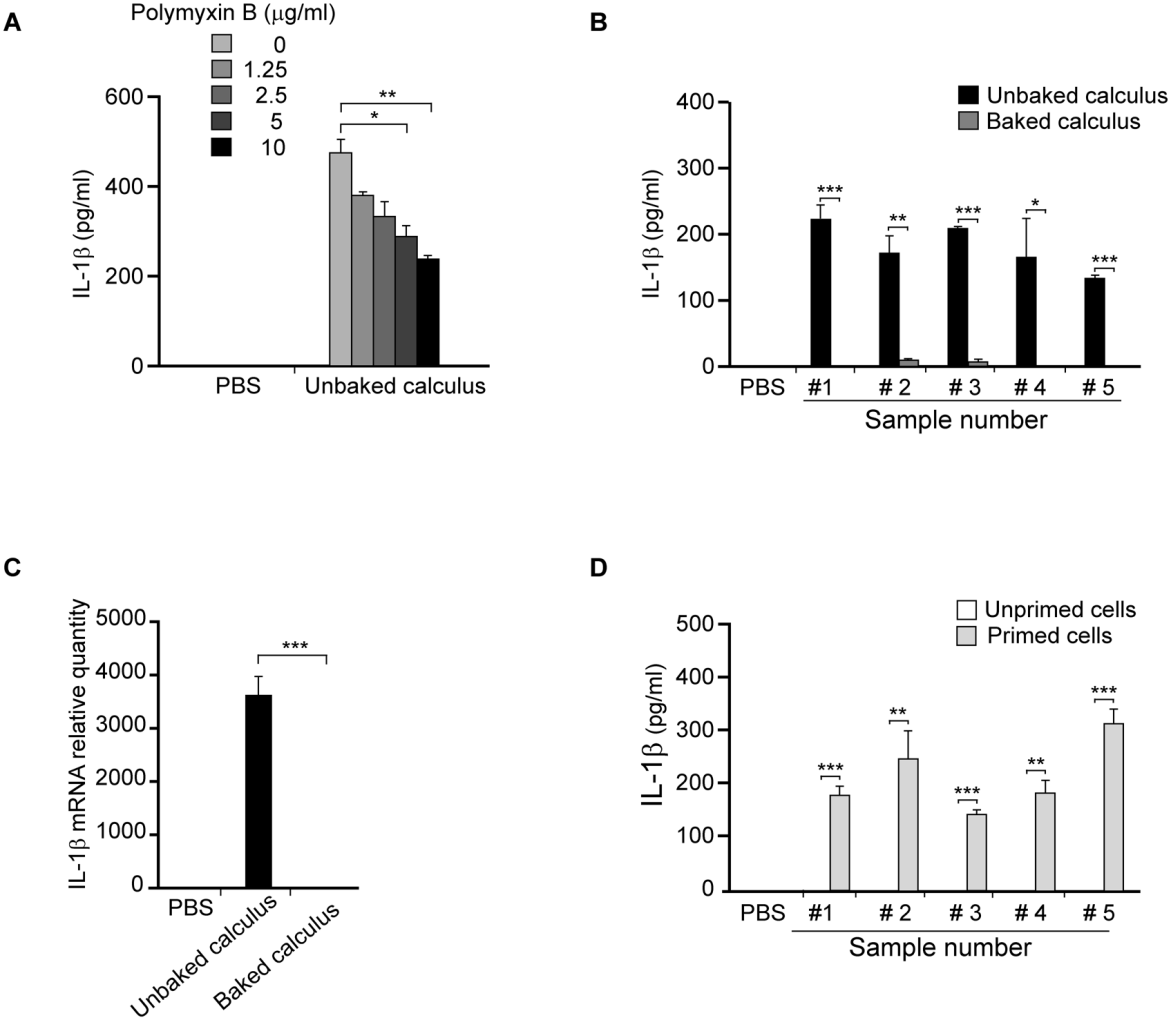
Effects of polymyxin B and dry-heat treatment of dental calculus on IL-1β secretion. (A) Macrophages from wild-type mice were pre-incubated with the indicated concentrations of polymyxin B for 30 min and either stimulated with 200 μg/mL unbaked calculus or left unstimulated (PBS). After 8 h of incubation, IL-1β concentrations in the culture supernatants were measured by ELISA. Representative results of three independent experiments are shown. (B) Dental calculus samples obtained from five periodontitis patients were treated at 250°C for 1 h (baked) or left untreated (unbaked). Macrophages from wild-type mice were stimulated with 500 μg/mL unbaked or baked calculus for 8 h, and IL-1β concentrations in the culture supernatants were measured by ELISA. (C) Macrophages from wild-type mice were stimulated with 500 μg/mL baked or unbaked calculus (sample #1) for 8 h, and total RNA was extracted. The relative quantity of IL-1β mRNA was determined by RT-qPCR. The IL-1β mRNA levels were normalized to GAPDH and the expression levels of the unstimulated control were adjusted to 1. (D) Macrophages from wild-type mice were primed with 100 ng/mL LPS or left unprimed, and stimulated with 500 μg/mL baked calculus prepared as described in (B). After 8 h of incubation, IL-1β concentrations in the culture supernatants were measured by ELISA. Results are expressed as mean ± standard error of triplicate assays. *p < 0.05; **p < 0.01; ***p < 0.001 (*t*-tests).

A wide array of microbial products, including LPS, can induce pro-IL-1β transcription [38]. To examine whether LPS is present in unbaked calculus, macrophages were stimulated with 200 μg/mL of unbaked calculus in the presence or absence of polymyxin B, which interacts with the lipid A portion of LPS. Polymyxin B somewhat reduced the secretion of IL-1β that was induced by the unbaked calculus (Fig 4A), indicating that LPS is one of the microbial components of dental calculus that induces IL-1β production.

We repeated the experiment with baked calculus to test the effects of inorganic structure of dental calculus. Each sample of unbaked calculus induced relatively high levels of IL-1β secretion in mouse macrophages, whereas those induced by the corresponding baked calculus samples were substantially lower (Fig 4B), suggesting that dry-heat treatment abrogated the capacity of the calculus to induce transcription of pro-IL-1β. To confirm that this reduced IL-1β secretion was the result of a reduction in pro-IL-1β transcription, mouse macrophages were stimulated with 500 μg/mL of unbaked or baked calculus (sample #1 only), and expression levels of IL-1β mRNA were determined by RT-qPCR. While unbaked calculus induced high levels of IL-1β mRNA, baked calculus induced negligible IL-1β mRNA production (Fig 4C).

**Fig 5:**
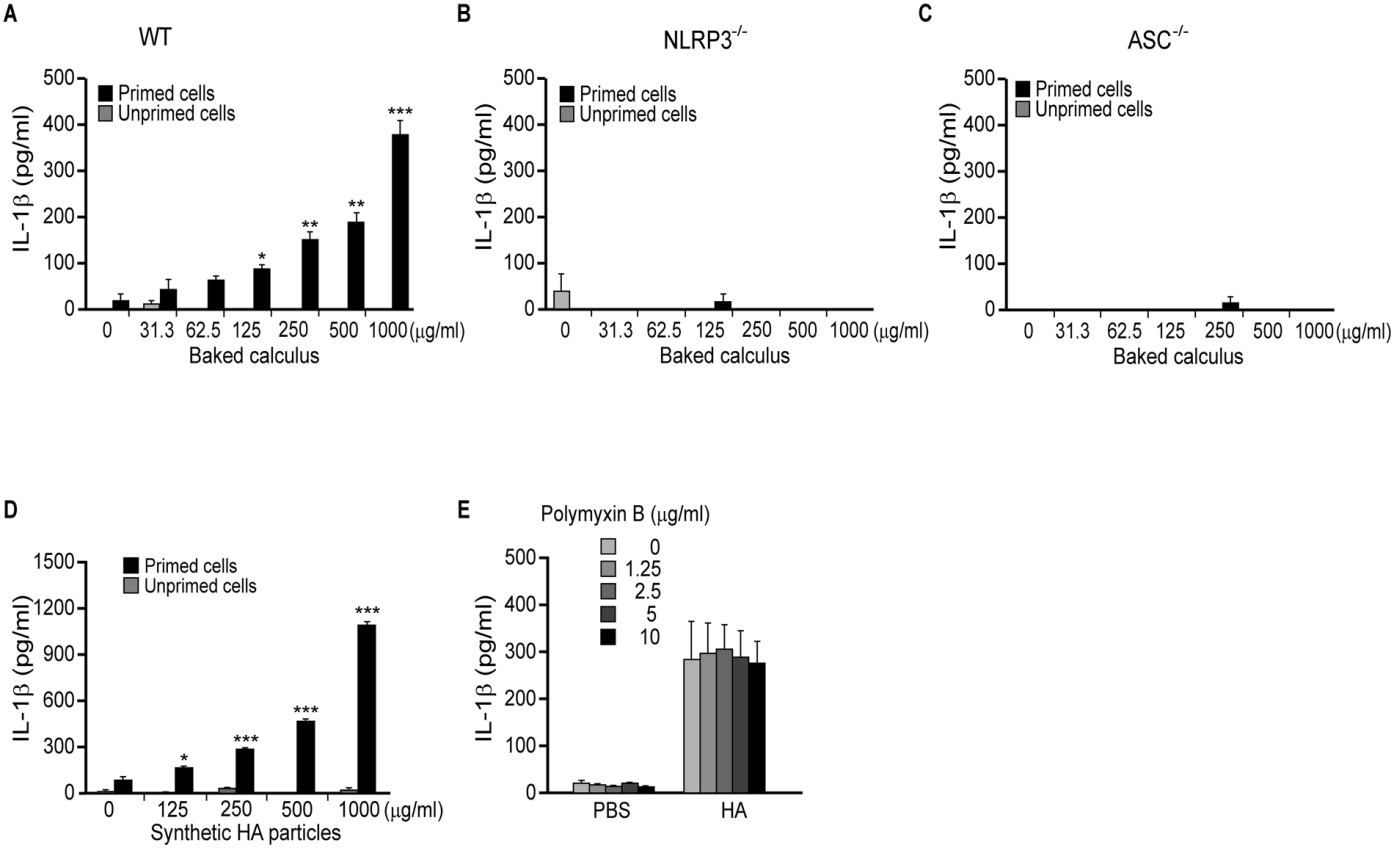
Induction of IL-1β by baked calculus and synthetic HA particles in lipid-A-primed mouse macrophages. (A-D) Macrophages from wild-type (A, D), NLRP3-deficient (B) and ASC-deficient (C) mice were primed with 200 ng/mL lipid A for 2 h, followed by stimulation with the indicated concentrations of baked calculus (A–C) or synthetic HA particles (≤ 5 μm) (D) for 8 h. IL-1β concentrations in the supernatants were measured by ELISA. The results are expressed as mean ± standard error of triplicate assays. Representative results of three independent experiments are shown. (E) Macrophages from wild-type mice were primed with 200 ng/mL lipid A for 2 h, pre-incubated with the indicated concentrations of polymyxin B for 30 min and stimulated with 500 μg/mL synthetic HA particles for 8 h. IL-1β concentrations in the supernatants were measured by ELISA. The results are expressed as mean ± standard error of three independent experiments. *p < 0.05; **p < 0.01; ***p < 0.001 (*t*-tests).

To examine whether the crystal structures of baked calculus induce inflammasome activation, mouse macrophages were primed with 100 ng/mL of LPS and stimulated with 500 μg/mL baked calculus. Each baked calculus sample induced IL-1β secretion in the primed macrophages, but not in unprimed cells (Fig 4D). Thus the crystalline structure of dental calculus can induce the inflammasome activation in mouse macrophages. Then, the macrophages from wild-type, NLRP3-deficient and ASC-deficient mice were primed with 200 ng/mL of lipid A and stimulated with increasing doses of baked calculus for 8 h. IL-1β was released in macrophages from wild-type mice in a dose-dependent manner (Fig 5A), whereas no IL-1β secretion was induced in macrophages from NLRP3-deficient and ASC-deficient mice (Fig 5B and 5C). Thus, IL-1β secretion induced by baked calculus was mediated by NLRP3 and ASC in mouse macrophages. To further investigate the role of the crystalline structure of dental calculus, we stimulated mouse macrophages with increasing doses of HA, which is one of the main crystal forms comprising dental calculus. HA crystals induced IL-1β secretion in mouse macrophages primed with lipid A in a dose-dependent manner, whereas unprimed mouse macrophages produced minimal levels of IL-1β (Fig 5D). Polymyxin B did not inhibit the IL-1β secretion induced by HA crystals, confirming that there was no endotoxin contamination in the HA crystals (Fig 5E).

**Fig 6:**
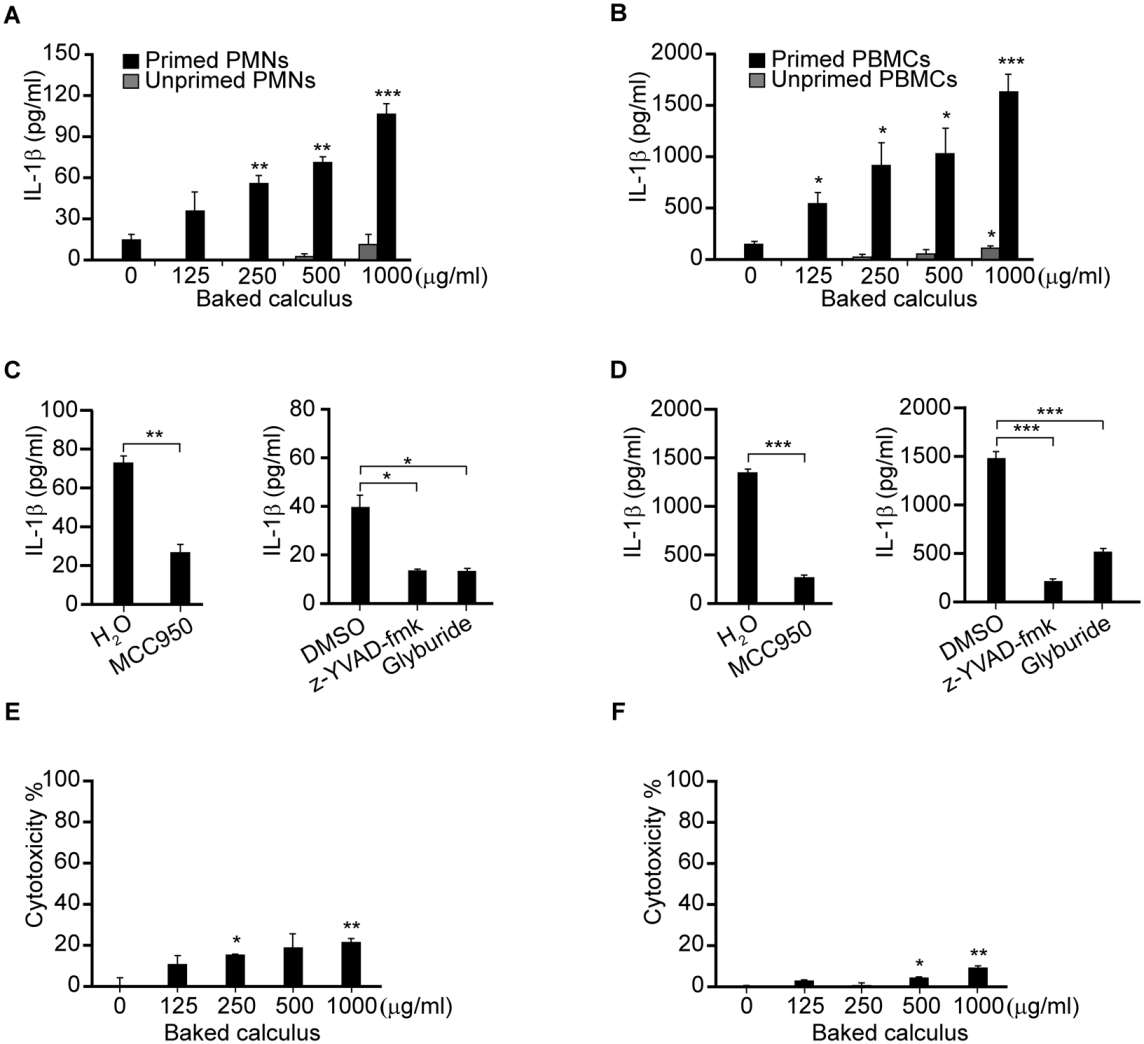
Baked calculus induces IL-1β secretion via NLRP3-inflammasome in primed human PMNs and PBMCs. Freshly isolated human PMNs (A, E) and PBMCs (B, F) were primed with 1 ng/mL lipid A and stimulated with the indicated concentrations of baked calculus for 6 h. PMNs (C) and PBMCs (D) were also primed with 1 ng/mL lipid A and stimulated with 500 μg/mL of unbaked calculus in the presence or absence of MCC950 (10 μM), z-YVAD-fmk (10 μM) or glyburide (25 μM). IL-1β concentrations in the supernatants were measured by ELISA. Cytotoxicity was quantified by measuring LDH release in the culture supernatants (E, F). The results are expressed as mean ± standard error of triplicate assays. Representative results of three independent experiments using different donors are shown. *p < 0.05; **p < 0.01; ***p < 0.001 (*t*-tests).

To investigate whether baked calculus induces NLRP3 inflammasome activation in human cells, human PMNs and PBMCs were primed with 1 ng/mL of lipid A and stimulated with increasing doses of baked calculus. IL-1β was produced in a dose-dependent manner in the PMNs (Fig 6A) and PBMCs (Fig 6B) that had been primed with lipid A, but not in unprimed cells. Preincubation with z-YVAD-fmk (10 μM), MCC950 (10 μM) or glyburide (25 μM) significantly reduced IL-1β secretion by PMNs (Fig 6C) and PBMCs (Fig 6D) stimulated with 500 μg/ml of baked calculus. These results indicate that the NLRP3 inflammasome is also involved in the IL-1β production induced by baked calculus in human PMNs and PBMCs. To investigate if baked calculus induces cell death, human PMNs and PBMCs were primed with 1 ng/mL of lipid A and stimulated with increasing doses of baked calculus. After 6 h of incubation, LDH release was induced in human PMNs (Fig 6E) and PBMCs (Fig 6F) in a dose-dependent manner. These results suggest that baked calculus induced the classical NLRP3 activation in human PMNs and PBMCs.

## Discussion

The stimulation of human PMNs and PBMCs with unbaked calculus resulted in the secretion of IL-1β, demonstrating that the unbaked calculus contained components that triggered both transcription and processing of IL-1β. This result was obtained despite the fact that the unbaked calculus had been extensively washed with 10% sodium hypochlorite and distilled water. Polymyxin B, which neutralizes LPS, reduced IL-1β secretion in macrophages, indicating that the endotoxin was one of the components of the dental calculus that induced the transcription of pro-IL-1β. It is likely that other bacterial ligands contained in the dental calculus also induced transcription of pro-IL-1β, since the treatment of dental calculus at 250°C for 1 h significantly reduced its capacity to induce pro-IL-1β transcription. Considering that this heat treatment cannot be performed in the oral cavity, it would be difficult to completely remove microbial components from calculus in situ. Even after extensive irrigation or administration of antibiotics, the calculus would still contain microbial remnants and therefore induce IL-1β production.

The secretion of IL-1β was inhibited by the caspase-1 inhibitor, z–YVAD-fmk, in all cells stimulated with unbaked calculus, indicating a caspase-1-dependent IL-1β secretion in both human and mouse phagocytes. The inflammasome was therefore involved in the IL-1β secretion that was induced by unbaked calculus. Inflammasomes are comprised of a member of the NLR family or AIM2, ASC, and caspase-1 [39]. Members of NLR family include NLRP1, NLRP3, and NLRC4, all of which may sense bacterial ligands contained in unbaked calculus. Bacterial muramyl dipeptide, pore-forming toxins, and flagellin are recognized by NLRP1, NLRP3, and NLRC4 respectively, while AIM2 recognizes bacterial dsDNA [40]. In addition, crystalline particles in unbaked calculus may also trigger the activation of NLRP3 inflammasome. We therefore examined which NLR family member was involved in inflammasome activation. We found that IL-1β secretion was significantly reduced by the NLRP3 inhibitors, MCC950 and glyburide, in human PMNs and PBMCs stimulated with unbaked calculus. In addition, IL-1β was not secreted in macrophages from NLRP3- or ASC-deficient mice stimulated with unbaked calculus, indicating that NLRP3 inflammasome is essential for the processing of IL-1β.

Extensive research has identified numerous stimuli that can activate the NLRP3 inflammasome. Among them is ATP, which acts through its cell surface receptor P2X7R [36]. Td92, a surface protein of the periodontopathogen *Treponema denticola*, activates NLRP3 through integrin α5β1 [25]. Nigericin acts as a pore-forming toxin to activate NLRP3. Crystalline particles, such as alum, silica, MSU, and basic calcium phosphate crystals also activate NLRP3 after phagocytosis [8]. Treatment with the phagocytosis inhibitor cytochalasin D attenuated IL-1β release in the cells stimulated with unbaked calculus, indicating that NLRP3 inflammasome activation by unbaked calculus is dependent on phagocytosis. This was consistent with the microscope analysis, which revealed a lower intake of dental calculus in the macrophages treated with cytochalasin D than in the untreated cells. Thus the soluble factors described above do not have a significant role in NLRP3 inflammasome activation in mouse macrophages. However, LPS may activate the alternative inflammasome pathway via TLR4 in human PMNs and PBMCs because as little as 7.8 μg/ml of unbaked calculus induced IL-1β production without significant levels of cytotoxicity in these cells.

Although we cannot exclude the possibility that other bacterial ligands, such as bacterial RNA:DNA hybrids, or new substances generated by heat treatment also play a part in activating NLRP3 inflammasome through phagocytosis in the cells stimulated with baked calculus [41], we assume that the crystal particles have a partial role. The finding that baked calculus induced IL-1β secretion in LPS-primed mouse macrophages supports this hypothesis. Furthermore, synthetic HA induced IL-1β secretion in lipid A-primed mouse macrophages, and baked calculus induced IL-1β in similarly primed human PMNs and PBMCs. These results suggest that the crystalline structure of dental calculus stimulates activation of NLRP3 inflammasome and contributes partially to IL-1β secretion in both human and mouse phagocytes. It is likely that baked calculus-induced NLRP3 activation was mediated by the classical inflammasome pathway because cytotoxicity was induced in parallel with IL-1β release in human PMNs and PBMCs.

In addition to the variety of bacterial ligands shown to induce IL-1β in dental plaque [42], the present findings revealed that dental calculus also stimulates IL-1β secretion. The production of IL-1β induced by dental calculus may have an additive effect on the inflammatory responses in periodontal tissue. In particular, when adequate plaque control has been achieved and microbial pathogens have been eliminated from periodontal pockets by periodontal surgery, retained dental calculus might be a major trigger of IL-1β. Persistent inflammation may therefore occur because calculus particles cannot be digested or removed by immune cells [43]. The inadequately removed dental calculus may also be a risk factor for complications of gingival graft surgery and implant surgery. Calculus can be attached to the apical root surface [44], which may have similar clinical significance to dental calculus attached to the root surface in periodontal pockets. Apical calculus may induce IL-1β release in human macrophages, and these deposits in periapical tissues may sustain apical periodontitis lesions indefinitely.

IL-1β release induced by dental calculus may promote inflammatory responses in periodontal tissues. The IL-1 receptor type 1 is expressed in almost all cell types in the periodontium [45]. Through the interaction with its receptor, IL-1β induces fibroblasts to synthesize collagenase and prostaglandin, and modulates the metabolism of connective tissue [46–48]. In gingival epithelial cells and fibroblasts, IL-6, IL-8, and MIP-1α are induced, which in turn induce migration and activation of leukocytes to promote inflammation [49, 50]. Furthermore, earlier studies identified purified IL-1 as a bone resorbing molecule involved in chronic inflammatory pathologies such as rheumatoid arthritis and periodontitis [51]. Later it was proven that recombinant IL-1 can stimulate bone resorption in vitro [52].

The present study is the first to show the capacity of dental calculus to induce IL-1β secretion in human and mouse phagocytes. Dental calculus is likely to contribute to the activation of NLRP3 inflammasome in periodontal tissue. It is noteworthy that the activation of NLRP3 inflammasome may lead to the processing of IL-18 and IL-33 [39, 53]. The effect of dental calculus on the production of these cytokines remains to be investigated. Studies using different cell types, such as gingival epithelial cells, are required to further understand how dental calculus affects periodontal tissue. Considering the effects of dental calculus, every attempt should be made to thoroughly remove calculus after initial preparation or surgical treatment. For this, the use of anti-tartar agents should be encouraged as part of routine periodontal therapy, and new strategies to remove dental calculus should be developed.

## Supporting Information

S1 Fig Inhibition of phagocytosis of unbaked calculus by cytochalasin D. Macrophages from wild-type mice were pre-incubated with 0 (A), 1 (B, D) or 7.5 μM (C) cytochalasin D and then incubated with 500 μg/mL unbaked calculus for 8 h. Light microscope images (A–C) and an electron microscope image (D) are shown. The white arrows mark the location of the unbaked calculus inside the cells, and black arrows mark the location of the unbaked calculus outside the cells. Scale bars: 20 μm (A–C); 5 μm (D).
(PDF)

S1 File Data set. This data set includes all relevant data of Figs 1–6.
(ZIP)
