# Supplementary figures and images for: Dental Calculus Stimulates Interleukin-1β Secretion by Activating NLRP3 Inflammasome in Human and Mouse Phagocytes
Source: PLoS One. 2016 Sep 15;11(9):e0162865. doi: 10.1371/journal.pone.0162865 (PMC5025015; doi:10.1371/journal.pone.0162865)

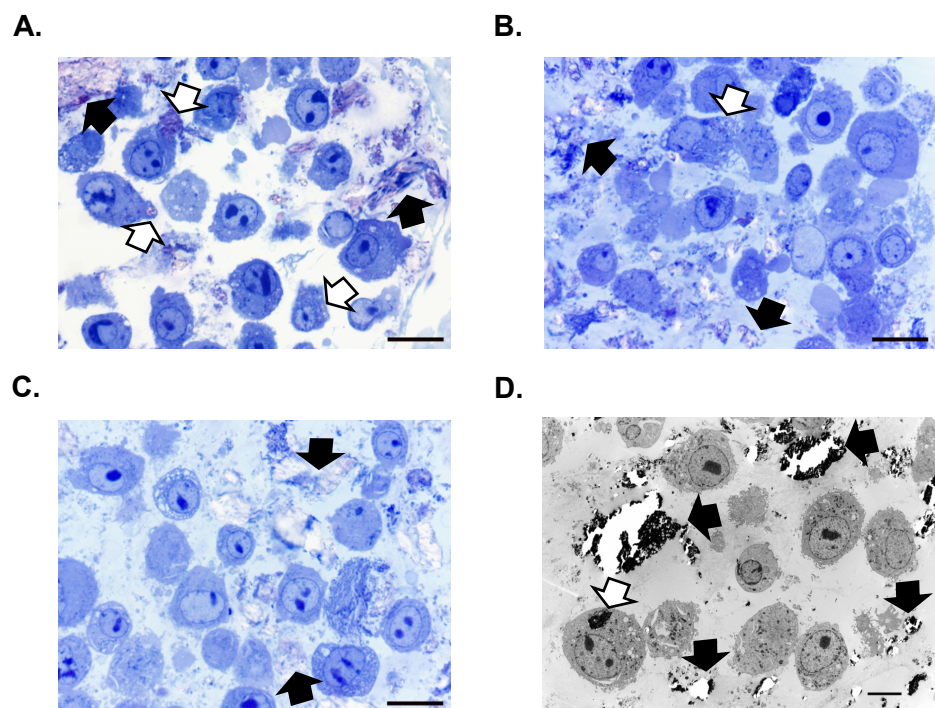

**S1 Fig. Inhibition of phagocytosis of unbaked calculus by cytochalasin D**

Supplement: S1 Fig — Macrophages from wild-type mice were pre-incubated with 0 (A), 1 (B, D) or 7.5 μM (C) cytochalasin D and then incubated with 500 μg/mL unbaked calculus for 8 h. Light microscope images (A–C) and an electron microscope image (D) are shown. The white arrows mark the location of the unbaked calculus inside the cells, and black arrows mark the location of the unbaked calculus outside the cells. Scale bars: 20 μm (A–C); 5 μm (D). (PDF) [file pone.0162865.s001.pdf]
